# *Cornus officinalis* Extract Ameliorates Fructose‐Induced Hepatic Steatosis in Mice by Sustaining the Homeostasis of Intestinal Microecology and Lipid Metabolism

**DOI:** 10.1002/fsn3.70425

**Published:** 2025-06-11

**Authors:** Liang Chen, Yingying Song, Yurou Huang, Junjie Hu, Yan Meng, Ming Yuan, Guohua Zheng, Xuanbin Wang, Cong Zhang, Zhenpeng Qiu

**Affiliations:** ^1^ School of Pharmacy, Hubei University of Chinese Medicine Wuhan China; ^2^ Laboratory of Chinese Herbal Pharmacology, Department of Pharmacy Renmin Hospital, Biomedical Research Institute, Hubei Key Laboratory of Wudang Local Chinese Medicine Research, Hubei University of Medicine Shiyan China; ^3^ College of Basic Medical Science, China Three Gorges University Yichang China; ^4^ Hubei Key Laboratory of Tumor Microenvironment and Immunotherapy, China Three Gorges University Yichang China; ^5^ Hubei Provincial Key Laboratory for Chinese Medicine Resources and Chinese Medicine Chemistry, Hubei University of Chinese Medicine Wuhan China; ^6^ Hubei Shizhen Laboratory Wuhan China; ^7^ Center of Traditional Chinese Medicine Modernization for Liver Diseases, Hubei University of Chinese Medicine Wuhan China

**Keywords:** *Cornus officinalis*, fructose, hepatic steatosis, intestinal microecology homeostasis, lipid metabolic disorder

## Abstract

*Cornus officinalis* Sieb. et Zucc. ( *Cornus officinalis* ), an edible natural plant fruit, has beneficial effects on a multitude of metabolic diseases, but the mechanism to improve hepatic steatosis remains elusive. In this study, the curative effect of *Cornus officinalis* extract (COE) is evaluated in a fructose‐induced NAFLD mouse model using biochemical indicators monitoring, histological staining, 16S rRNA sequencing analysis, and fecal microbiota transplantation. Our results showed that COE attenuates hepatic steatosis in fructose‐fed mice. Mechanistically, COE repairs intestinal barrier damage and gut flora dysbiosis to suppress proinflammatory microbe‐derived metabolite transportation to the liver, thus inhibiting the hepatic inflammation and lipid metabolic dysfunction. Notably, transplantation of fecal microbiota isolated from the fructose‐fed mice could reverse the beneficial effect of COE on attenuating NAFLD. Therefore, our study demonstrates that COE delays the progression of fructose‐driven NAFLD by suppressing lipid metabolic dysfunction and gut microbiota‐mediated liver inflammation, highlighting the potential of *C. officinalis* as a resource for the treatment of NAFLD drugs.

## Graphical abstract

Our study demonstrates that *Cornus officinalis* ethanol extract delays the progression of fructose‐driven NAFLD by suppressing lipid metabolic dysfunction and gut microbiota‐mediated liver inflammation. Our study provides a novel potential strategy that dietary supplementation with *C. officinalis* might be a promising strategy for NAFLD treatment, especially for patients with diets of excessive fructose consumption.

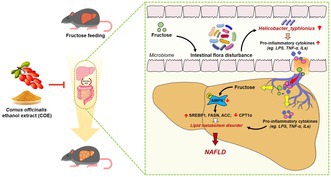

**Abbreviations**
ACC: acetyl CoA carboxylase
ALT: alanine aminotransferase
AST: aspartate aminotransferase

*Cornus officinalis*

:
*Cornus officinalis* Sieb. et Zucc.
COE:
*Cornus officinalis* extract
CPT1α: carnitine palmityl transferase 1α
FASN: fatty acid synthase
HDL‐c: high‐density lipoprotein cholesterol
IL‐1β: interleukin‐1β
IL‐18: interleukin‐18
IL‐6: interleukin‐6
LDL‐c: low‐density lipoprotein cholesterol
LPS: lipopolysaccharide
MDA: malondialdehyde
NAFLD: non‐alcoholic fatty liver disease
ROS: reactive oxygen species
SREBP‐1: sterol‐regulatory element binding protein‐1
TC: total cholesterol
TG: triglyceride
TNF‐α: tumor necrosis factor‐α
T‐SOD: total superoxide dismutase
ZO‐1: zonula occludens‐1

## 1 Introduction

Non‐alcoholic fatty liver disease (NAFLD), recently renamed metabolic dysfunction‐associated fatty liver disease, affects almost one‐quarter of the global population with a steadily increasing and younger incidence (Goldner and Lavine 2020; Teng et al. 2023). Most patients with NAFLD are at risk of progression to steatohepatitis, fibrosis, cirrhosis, and ultimately hepatocellular carcinoma. Thus, NAFLD has become a leading cause of liver‐related morbidity and mortality, imposing a significant burden on global public health (Lee et al. 2023). However, no definitively effective medications have been identified for its clinical treatment until now, highlighting the need for further research to develop practical strategies for NAFLD prevention and therapy.

Although excessive lipid deposition in adipocytes is widely considered the central abnormality initiating NAFLD, multiple factors, including the interaction between unhealthy dietary and systematic metabolism, are involved in its progression (Yang et al. 2024). With the secular changes in modern dietary habits, fructose is increasingly consumed as sucrose or high‐fructose corn syrup in fruits, soft drinks, and other sugar‐sweetened foods, becoming a sweet burden on human health, particularly in the etiology of metabolic diseases like NAFLD (Alwahsh and Gebhardt 2017; Jensen et al. 2018). Dietary fructose was initially thought to be metabolized exclusively in the liver, where it converts acetyl coenzyme A to fatty acids by promoting fatty acid *de novo* synthesis. Currently, fructose metabolism is also recognized in the small intestine, where intestinal microbes contribute to its absorption, providing substrates for subsequent hepatic fatty acid synthesis (Taylor et al. 2021; Zhao et al. 2020). This highlights the critical role of gut–liver crosstalk in dietary fructose metabolism. Substantial evidence has indicated that gut–liver axis dysfunction plays an important role in fructose‐driven NAFLD, including imbalanced gut microbiota and compromised intestinal barrier function (Vasques‐Monteiro et al. 2021). Moreover, overconsumption and abuse of fructose in the small intestine trigger intestinal barrier deterioration, leading to bacterial translocation and endotoxemia (Cho et al. 2021). Disruption of gut epithelial integrity increases intestinal permeability, exposing the liver to the systemic circulation of toxins and bacterial dissemination. Consequently, hepatic metabolic disorders and disease processes accelerate via the gut‐liver axis (Leung et al. 2016; Todoric et al. 2020). Emerging evidence suggests that restoring intestinal barrier integrity and balancing gut microbiota can alleviate NAFLD (Brandt et al. 2019; He et al. 2022; Li et al. 2023). Therefore, intestinal microbial imbalance, hepatic fructose metabolism, and their gut‐liver dialogue are central to fructose‐driven NAFLD. Maintaining gut‐liver functional homeostasis might thus serve as a prospective strategy to delay NAFLD progression.

In recent years, with the emergence of the “food‐as‐drug” concept (Mafra et al. 2021), especially as it is closely related to metabolic diseases, there is increasing interest in exploring pharmacologically active natural molecules of food origin as potential therapeutic agents to halt the progression of NAFLD. *Cornus officinalis* Sieb. et Zucc. ( *Cornus officinalis* ), an edible natural plant fruit, has beneficial effects on a multitude of metabolic diseases in Chinese medicine clinics (Gao et al. 2021; Huang et al. 2018), which can serve as raw materials for the development of green health food, such as beverages, jams, and preserves (Czerwinska et al. 2021). Modern pharmacological studies have shown that *C. officinalis* and its different medical forms have beneficial effects on a multitude of metabolic diseases (Cao et al. 2022; Han et al. 2014; Park et al. 2021). Meanwhile, there have been reports suggesting its effect on correcting dyslipidemia and reducing hepatic lipid deposition (Cao et al. 2022; Park et al. 2021). The above studies suggest that *C. officinalis* may be beneficial in the treatment of NAFLD. However, the effect of *C. officinalis* on improving NAFLD by maintaining gut‐liver function homeostasis has not been reported yet. Here, a fructose‐fed mouse model was established to investigate the efficacy and mechanism of *C. officinalis* extract (COE) in delaying NAFLD by targeting the gut–liver axis. Our study demonstrates that COE ameliorates fructose‐induced NAFLD by restoring intestinal flora homeostasis and lipid metabolism disorder. This finding reveals a novel potential strategy that dietary supplementation with *C. officinalis* might be a promising strategy for NAFLD treatment, especially for patients with diets of excessive fructose consumption.

## 2 Materials and Methods

### 2.1 Materials

Fructose (purity ≥ 99%) was bought from Aladdin Biochemical Technology Co. Ltd (Shanghai, China). Commercial kits for biochemical detection, such as triglyceride (TG), total cholesterol (TC), low‐density lipoprotein cholesterol (LDL‐c), high‐density lipoprotein cholesterol (HDL‐c), alanine aminotransferase (ALT), aspartate aminotransferase (AST), reactive oxygen species (ROS), malondialdehyde (MDA), and total superoxide dismutase (T‐SOD) were obtained from Jiancheng Bioengineering Institute (Nanjing, China). ELISA kits for the detection of lipopolysaccharide (LPS), tumor necrosis factor‐α (TNF‐α), interleukin‐6 (IL‐6), IL‐1β, and IL‐18 were purchased from Ruixin Biotechnology Co. Ltd (Quanzhou, China). All other reagents used were of analytical grade.

### 2.2 COE Preparation

*Cornus officinalis* collected in Anhui province (China) was purchased from Beijing Shizhentang (Yichang) Pharmaceutical Co., LTD (Yichang, China) and authenticated by Prof. Guohua Zheng (School of Pharmacy, Hubei University of Chinese Medicine, China). COE was prepared as described previously (Quah et al. 2020). Briefly, *C. officinalis* (500 g) was ground into fine microparticles using a powder machine, and then filtered through a 100‐mesh sieve. The powder (100 g) was dissolved in 75% ethanol at a volume ratio of 1:10 under ultrasonic conditions for 60 min and continuously stirred during the extractive process, followed by concentrating to approximately 200 mL on a rotary evaporator, then lyophilized with a freeze‐dryer to obtain dried COE powder and preserved under a dry conditions at room temperature.

### 2.3 Analysis of Phytochemical Constituents in COE

#### 2.3.1 High Performance Liquid Chromatography (HPLC) Analysis

An Agilent 1260 HPLC system (Agilent Technologies, Palo Alto, USA) was used to analyze the main phytochemicals in the COE. 0.3% phosphoric acid (A) and acetonitrile (B) were used as the mobile phase at a flow rate of 1.0 mL/min. The elution procedure was optimized as follows: 0–5 min, 10% B; 6–20 min, 10% ~ 60% B; 21–30 min, 60% ~ 80% B. 10 μL sample was injected into a Symmetry C18 reverse‐phase column (4.6 mm × 250 mm, 5 μm) at 30°C, and the UV–vis detection wavelength was 240 nm.

#### 2.3.2 Ultra‐Performance Liquid Chromatography Quadrupole Time of Flight Mass Spectrometry (UPLC‐Q‐TOF‐MS) Analysis

An UHPLC‐Q‐TOF‐MS system with MassHunter MS workstation and METILIN database was used to analyze phytochemical constituents in COE as previously described (Chen, Liu, et al. 2022). 0.3% phosphoric acid (A) and acetonitrile (B) were used as the mobile phase at a flow rate of 0.3 mL/min. The elution procedure was optimized as follows: 0–5 min, 5%–10% B; 6–20 min, 10%–60% B; 21–30 min, 60%–80% B. COE methanol solution was injected at a flow rate of 0.3 mL/min into an InfinityLab Poroshell 120 EC single bond C18 column (3.0 × 150 mm, 2.7 μm) at 30°C. Mass data acquisition was performed with an Agilent 6530 Q‐TOF (Agilent Technologies, Palo Alto, USA). Phytochemical constituents in COE were identified using the METLIN library (https://metlin.scripps.edu).

### 2.4 Animal Monitoring

Male C57BL/6 mice (weight 20 ± 2 g) were obtained from the Center of Experimental Animals of Hubei Province (Wuhan, China). All mice were randomly ranged into five groups (*n* = 8 per group) and fed standard mouse chow. The control group was allowed free access to plain water, and the fructose diet group and administration groups were fed the 30% fructose solution. Meanwhile, the administration groups were given COE (100 or 200 mg/kg) by gavage daily for a continuous 8 weeks (Park et al. 2020). Fenofibrate served as a positive control drug in this study by gastric administration with a dose of 100 mg/kg in fructose‐fed mice (Shin et al. 2021). At the end of the animal experiments, all mice were sacrificed with 3% isoflurane to collect serum, liver specimens, and intestinal contents for the following investigations. Animal use procedures in this study were conducted in accordance with the Guidelines for the Management and Use of Laboratory Animals (original edition 8, National Council for Academic Research, 2012) and were approved by the Institutional Animal Care and Use Committee of Hubei University of Chinese Medicine (HUCMS202106003).

### 2.5 Hematoxylin–Eosin, Oil Red O, and Periodic Acid‐Schiff Staining

Fresh liver tissues and intestinal samples were fixed with 10% paraformaldehyde, dehydrated, embedded with paraffin, and then cut into 5 μm slices. Paraffin liver and intestinal sections were processed for xylene dewaxing and gradient ethanol rehydration and subsequently stained with hematoxylin–eosin (H&E) dye. The hepatic steatosis scoring system consisted of a semi‐quantitative assessment of three histological features: steatosis (0–3), lobular inflammation (0–3), and hepatocellular ballooning (0–2) (Kleiner et al. 2005). Intestinal sections were also subjected to periodic acid‐Schiff (PAS) staining using PAS dye. For oil Red O (ORO) staining, frozen liver slices were stained with ORO solution, rinsed with 60% isopropanol, and then redyed nuclei with hematoxylin. All these segments were mounted under coverslips for further microscopic visualization.

### 2.6 Western Blotting, Immunohistochemistry, and Immunofluorescence

Cytosolic proteins were extracted from liver and intestinal tissues using an M‐PER lysate buffer mixed with protease inhibitor and phosphatase inhibitor, respectively. The concentrations of extracted protein were determined using a BCA kit, and protein samples were then prepared after adding the loading buffer and heated at 95°C for 5 min to allow protein denaturation. Denatured proteins were separated with SDS‐PAGE and semi‐dry transferred onto the PVDF membrane. After blocking for at least 1 h in blocking buffer, the membranes were incubated with specific primary antibodies overnight, followed by incubation with corresponding secondary antibodies. The protein bands were then assessed by the chemiluminescent substrate and quantified using Image J 1.51 software.

The immunohistochemical assay was performed using an SP Rabbit & Mouse HRP Kit following the manual. In brief, after dewaxing and rehydration, antigen retrieval was performed on liver sections in boiling sodium citrate buffer, then cooled to room temperature. Sections were incubated with primary antibodies overnight at 4°C, then the immunoreactivity was observed using the DAB stain and captured under a microscope.

For immunofluorescent staining, antigen‐retrieved intestinal slices were incubated with primary antibodies overnight and FITC‐conjugated secondary antibodies. The region of fluorescence intensity was examined and imaged using an Olympus IX 73 DP80 fluorescence microscope (Tokyo, Japan). Cell nuclei were stained with blue (DAPI). All involved antibodies were listed in Table S1.

### 2.7 Real‐Time Quantitative Polymerase Chain Reaction

Total RNA was extracted from liver and intestinal homogenates with TRIzol Reagent, respectively. After that, complementary DNA (cDNA) was obtained through reverse transcription using M‐MLV reverse transcriptase and oligo‐dT18 primer. The real‐time quantitative polymerase chain reaction (RT‐qPCR) assay was then processed via FastStart Universal SYBR Green Master Mix on the ABI StepOne PlusTM PCR System (Applied Biosystems, Foster City, CA). Relative mRNA levels were normalized according to the GAPDH reference. All primer sequences were listed in Table S2.

### 2.8 Reactive Oxygen Species Assay

Reactive Oxygen Species (ROS) generation was determined using flow cytometry. In detail, primary hepatocytes were isolated from fresh liver suspension, then stained with 2′‐7′‐dichlorofluorescin diacetate (DCFH‐DA) at 37°C for 30 min and washed with PBS. After centrifugation, the supernatant was discarded to collect cells, and the cells were resuspended in 0.5 mL PBS solution, followed by analysis with a flow cytometer.

### 2.9 16S rRNA Sequencing Analysis

Fecal samples were collected from the control or fructose‐fed mice with intragastric administration of either vehicle or COE (200 mg/kg) for 8 weeks, respectively. The total DNA was extracted from the fecal samples using Stool Genomic DNA Extraction Kits (Solarbio, Beijing, China). Primers (forward: 5′‐ACTCCTACGGGAGGCAGCA‐3′ and reversed: 5′‐GGACTACHVGGGTWTCTAAT‐3′) were used to amplify the 16S rRNA gene in the V3‐V4 region. Further, the PCR products were purified and quantified to establish a sequencing library, and the qualified libraries were sequenced using the Illumina Miseq platform (Illumina NovaSeq 6000, Illumina, San Diego, CA, USA) according to the manufacturer's instructions. Finally, the changes in microbial communities between different groups were evaluated by various analyses (including OTU analysis, α/β diversity analysis, structure and abundance analysis, etc.) as previously described (Chen, Wang, et al. 2022).

### 2.10 Fecal Microbiota Transplantation

Fecal microbiota transplantation was performed as previously described (Peng et al. 2023). Briefly, male C57BL/6 mice were randomly divided into four groups (*n* = 6 per group). Mice in the control group were allowed free access to water, and fructose diet groups were fed with a 30% fructose solution for 8 weeks. In the meantime, intragastrical administrations of COE (200 mg/kg) were performed daily in the treatment groups. After 4 weeks, the feces from fructose‐diet mice were collected daily in sterile tubes with normal saline (200 mg/mL) for homogenization and then centrifuged at 2000 g/min for 1 min to collect the supernatant to obtain the fecal microbiota solution. Mice in the fecal microbiota transplantation group were administered intragastrically with the fecal microbiota solution (0.2 mL/day) in addition to COE daily. Finally, an evaluation of the role of intestinal flora in the ameliorative effect of COE in fructose‐fed mice was processed by observing alterations in liver histology and lipid metabolism function.

### 2.11 Statistical Analysis

Prism 8.0 (GraphPad Software Inc., San Diego, CA, USA) was used for statistical analysis. All data were represented as Mean ± SD values. Two‐tailed unpaired *t*‐test and ANOVA were performed between two groups and among three or more groups, respectively. *p* < 0.05 means statistical significance.

## 3 Results

### 3.1 Qualitative Analysis of Phytochemical Constituents in COE

A UPLC‐QTOF‐MS method was used to identify the major phytochemical constituents in COE. A total of 31 components were identified in the positive and negative modes using the METLIN library (https://metlin.scripps.edu) (Table S3). Among them, 5 representative compounds of COE were respectively characterized as morroniside, loganin, cornuside I, gallic acid, and magnolin by HPLC analysis (Figure S1). It can be concluded that COE was enriched with iridoid glycosides, which were consistent with previous studies (Huang et al. 2018).

### 3.2 COE Alleviates Fructosecinduced Hepatic Steatosistosimice Mice

**FIGURE 1:**
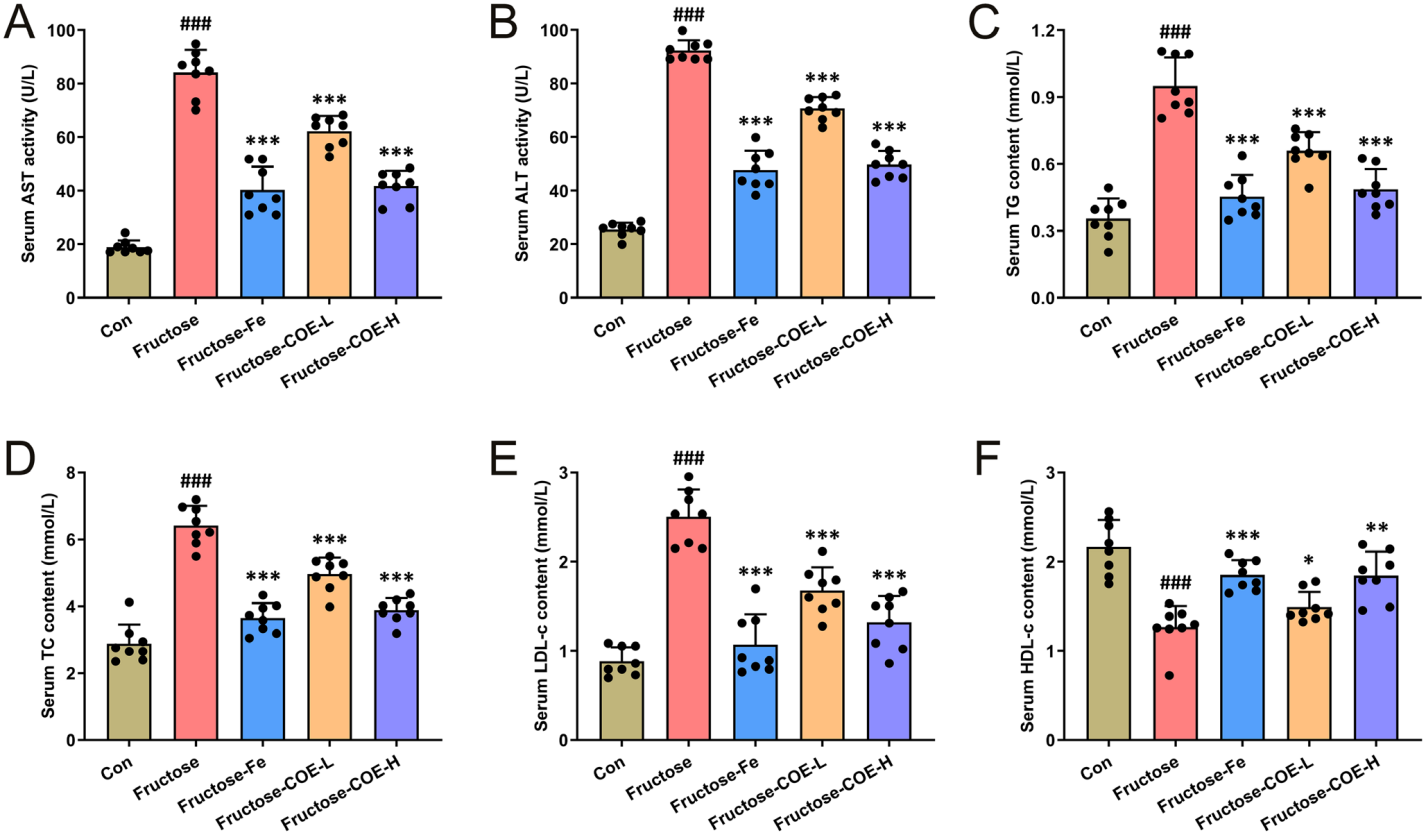
*Cornus officinalis* extract (COE) normalizes liver functional and lipid metabolic indices in fructose‐fed mice. (A, B) Activity of AST (A) and ALT (B). (C–F) Serum levels of TG (C), TC (D), LDL‐c (E), and HDL‐c (F). Mean ± SD, *n* = 8. ^###^ *p* < 0.001 versus the Con group; *p < 0.05, **p < 0.01, ***p < 0.001 versus the fructose‐induced group. COE‐L and COE‐H represent intragastric administration of *Cornus officinalis* extract at low (100 mg/kg) and high (200 mg/kg) doses, respectively.

**FIGURE 2:**
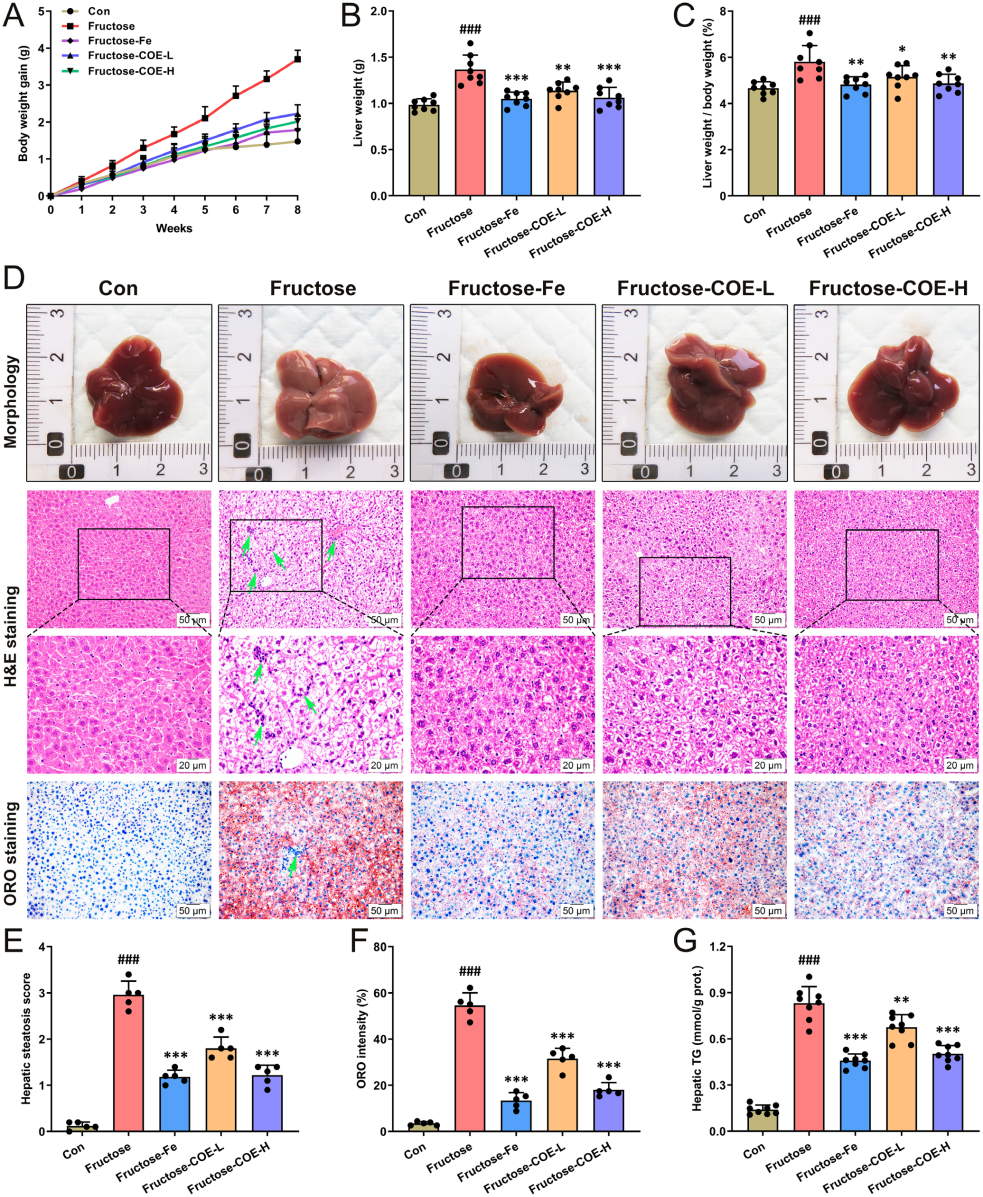
*Cornus officinalis* extract (COE) alleviates fructose‐induced hepatic steatosis in mice. (A) body weight gain. (B, C) Liver weight (B) and the ratio of liver weight to body weight (C). (D) Morphological appearance and representative H&E and ORO staining of the liver samples from the Con or fructose‐fed mice with intragastric administration of either vehicle or COE for 8 weeks, respectively (original magnification: 200×; scale bar: 50 μm; 400×, scale bar: 20 μm). (E, F) Hepatic steatosis scores (E) and hepatic ORO intensity (F). (G) TG content in liver tissue samples. Mean ± SD, *n* = 8. ^###^ *p* < 0.001 versus the Con group; *p < 0.05, **p < 0.01, ***p < 0.001 versus the fructose‐induced group. COE‐L and COE‐H represent intragastric administration of *Cornus officinalis* extract at low (100 mg/kg) and high (200 mg/kg) doses, respectively.

Since its wide utilization in daily diet nowadays, fructose is becoming a critical independent risk factor of NAFLD that deserves more attention (Febbraio and Karin 2021). In this study, a fructose‐driven hepatic steatosis mouse model was constructed by feeding with fructose solution for continuous 8 weeks, and simultaneous administration of COE by gavage (100 or 200 mg/kg) was performed to investigate the effect of COE on fructose‐induced NAFLD. Our results showed that the activities of liver function enzymes (AST and ALT) were increased in fructose‐fed mice while decreased after COE treatment (Figure 1A,B). The serum contents of TG, TC, and LDL‐c were also upregulated but HDL‐c was reduced in fructose‐fed mice, which were reversed by COE (Figure 1C–F). Moreover, the body weight, liver weight, and liver index were increased in fructose‐fed mice (Figure 2A–C), while they were decreased by COE administration. Morphologically, the livers of fructose‐fed mice were pale yellow but tended to be normal in COE‐treated mice (Figure 2D). Histological staining showed vesicular steatosis, lipid accumulation, and inflammatory infiltration (green arrow) in the liver of fructose‐fed mice along with elevated hepatic steatosis score, ORO intensity and hepatic TG content, which were improved by COE administration (Figure 2D–G). Compared with the positive effect of fenofibrate (Fe), a hypolipidemic medicine, it showed that COE displayed a comparable effect in improving liver dysfunction, dyslipidemia, and steatosis. All these reveal that COE is effective in suppressing NAFLD progression in fructose‐fed mice.

### 3.3 COE Sustains Hepatic Lipid Metabolism Homeostasis in Fructose‐Fed Mice

**FIGURE 3:**
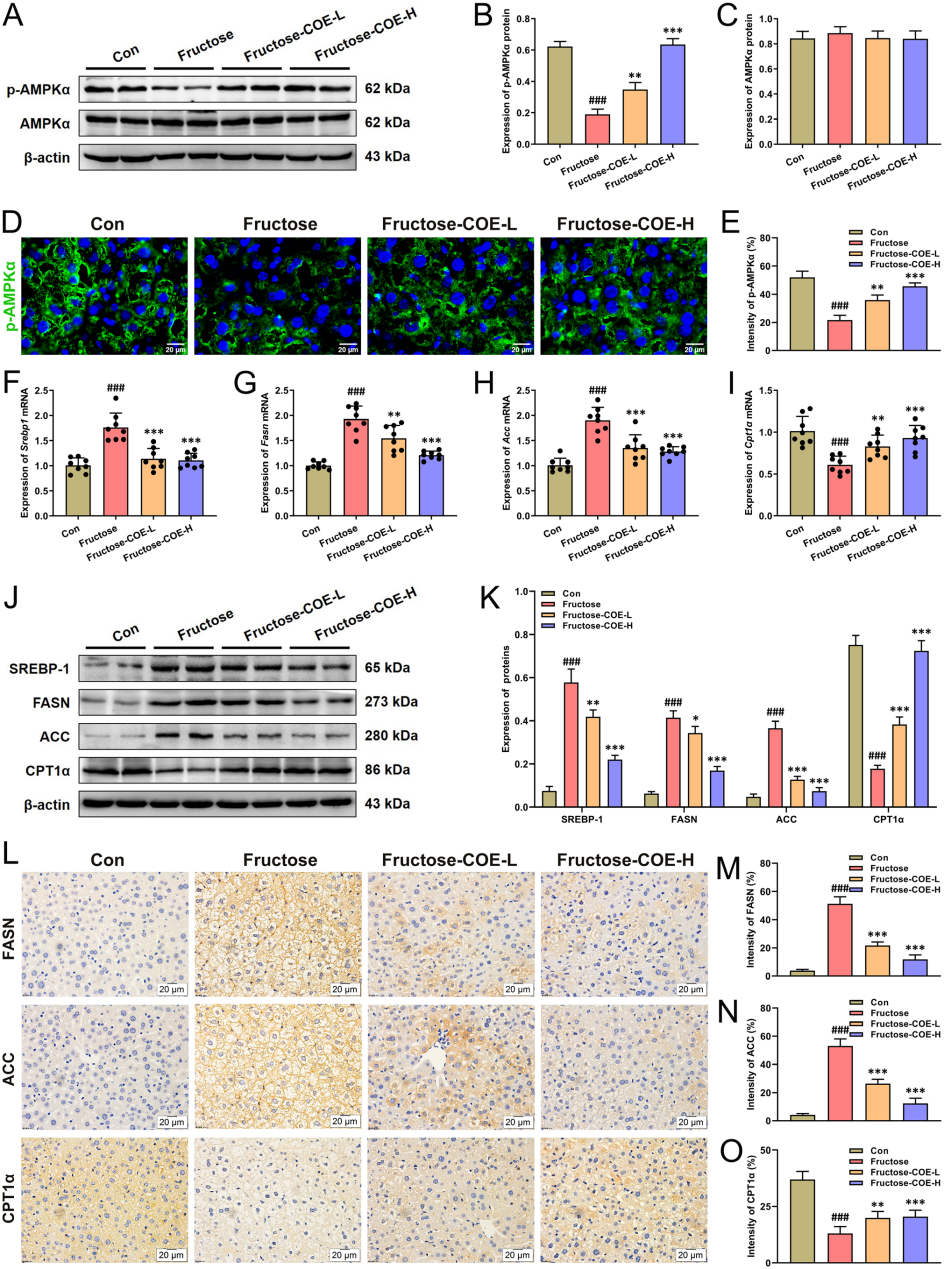
*Cornus officinalis* extract (COE) improves fructose‐induced hepatic lipid dysregulation in mice. (A‐C) Representative immunoblot images and quantitative analysis of optical density for p‐AMPKα and AMPKα protein expression in the liver. (D, E) Representative immunofluorescence images and quantitative analysis of optical density for p‐AMPKα in the liver. (F–I) The mRNA expression levels of *Srebp1*, *Fasn*, *Acc*, and *Cpt1α* in the liver. (J, K) Representative immunoblot images and quantitative analysis of SREBP‐1, FASN, ACC, and CPT1α protein expression. β‐Actin confirms loading in immunoblotting. (L–O) Representative immunohistochemistry images and quantitative analysis of optical density for FASN, ACC, and CPT1α protein expression. Mean ± SD, *n* = 8 for mRNA expression analysis, *n* = 3 for protein expression analysis. ^###^ *p* < 0.001 versus the Con group; *p < 0.05, **p < 0.01, ***p < 0.001 versus the fructose‐induced group. COE‐L and COE‐H represent intragastric administration of *Cornus officinalis* extract at low (100 mg/kg) and high (200 mg/kg) doses, respectively.

Lipid metabolism homeostasis is known to be important in the development of NAFLD. Here, the fructose diet was found to disrupt the balance of hepatic lipid metabolism, whereby lipid synthesis was enhanced but oxidation was diminished. In detail, the results showed that fructose feeding suppressed the phosphorylation of AMPK, the key factor of fructose‐induced hepatic lipid metabolism disorder (Woods et al. 2017), while COE treatment promoted its activation (Figure 3A–C). Similar results were found in immunofluorescence analysis (Figure 3D,E). In addition, both the mRNA and protein levels of key lipogenic factors (SREBP‐1, FASN, and ACC) were increased and the important molecule of fatty acid oxidation (CPT1α) was decreased in the liver of fructose‐fed mice, while reversed by COE administration (Figure 3F–K). Similar results were observed in immunohistochemical analysis (Figure 3L–O). Altogether, it suggests that COE can inhibit fructose‐induced lipogenesis and promote β‐oxidation to maintain lipid metabolism homeostasis in mice.

### 3.4 COE Suppresses Oxidative Stress and Inflammatory Response in the Liver of Fructose‐Fed Mice

**FIGURE 4:**
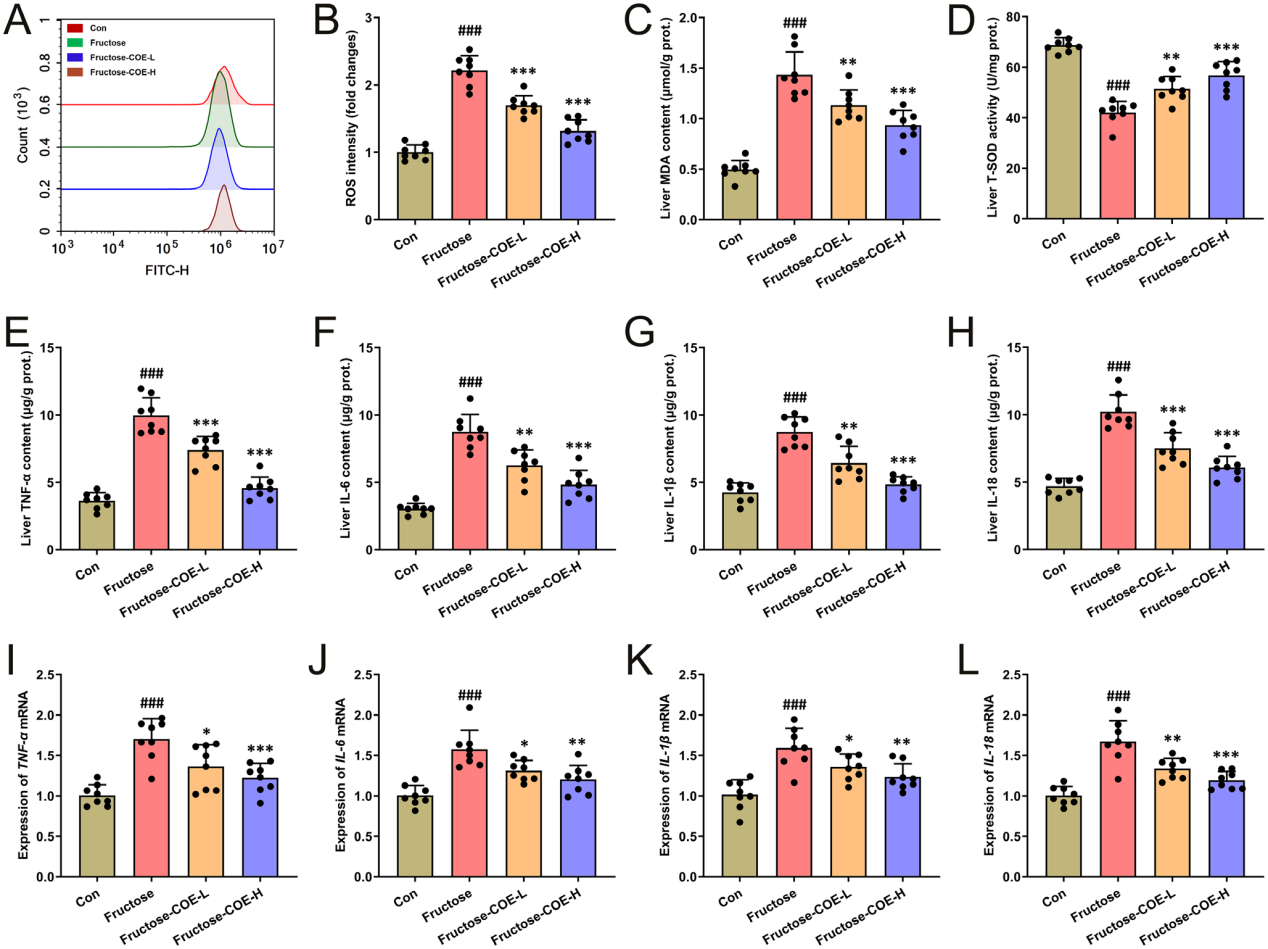
*Cornus officinalis* extract (COE) suppresses oxidative stress and inflammatory response in the liver of fructose‐fed mice. (A, B) Quantitative analysis of ROS fluorescence intensity. (C, D) MDA content (C) and T‐SOD activity (D) in the liver. (E–H) The mRNA expression levels of TNF‐α, IL‐6, IL‐1β, and IL‐18 in the liver. (I–L) Contents of the pro‐inflammatory factors TNF‐α, IL‐6, IL‐1β, and IL‐18 in the liver. Mean ± SD, *n* = 8, ^###^ *p* < 0.001 versus the Con group; *p < 0.05, **p < 0.01, ***p < 0.001 versus the fructose‐induced group. COE‐L and COE‐H represent intragastric administration of *C. officinalis* extract at low (100 mg/kg) and high (200 mg/kg) doses, respectively.

Given the lipid accumulation in hepatocytes and damaged liver function as above found, it might result in hepatic oxidation and inflammatory response. Coherently, it was found that hepatic ROS levels were increased in fructose‐fed mice while reduced by COE treatment (Figure 4A,B) accompanied by downregulated MDA content and elevated T‐SOD activity (Figure 4C,D). Moreover, the elevated contents and mRNA levels of pro‐inflammatory cytokines (TNF‐α, IL‐6, IL‐1β, and IL‐18) in the liver of fructose‐fed mice were also reduced after COE treatment (Figure 4E–L). These results indicate that COE is capable of inhibiting hepatic oxidative stress and inflammatory response in fructose‐fed mice.

### 3.5 COE Improves Intestinal Barrier Damage in Fructose‐Fed Mice

**FIGURE 5:**
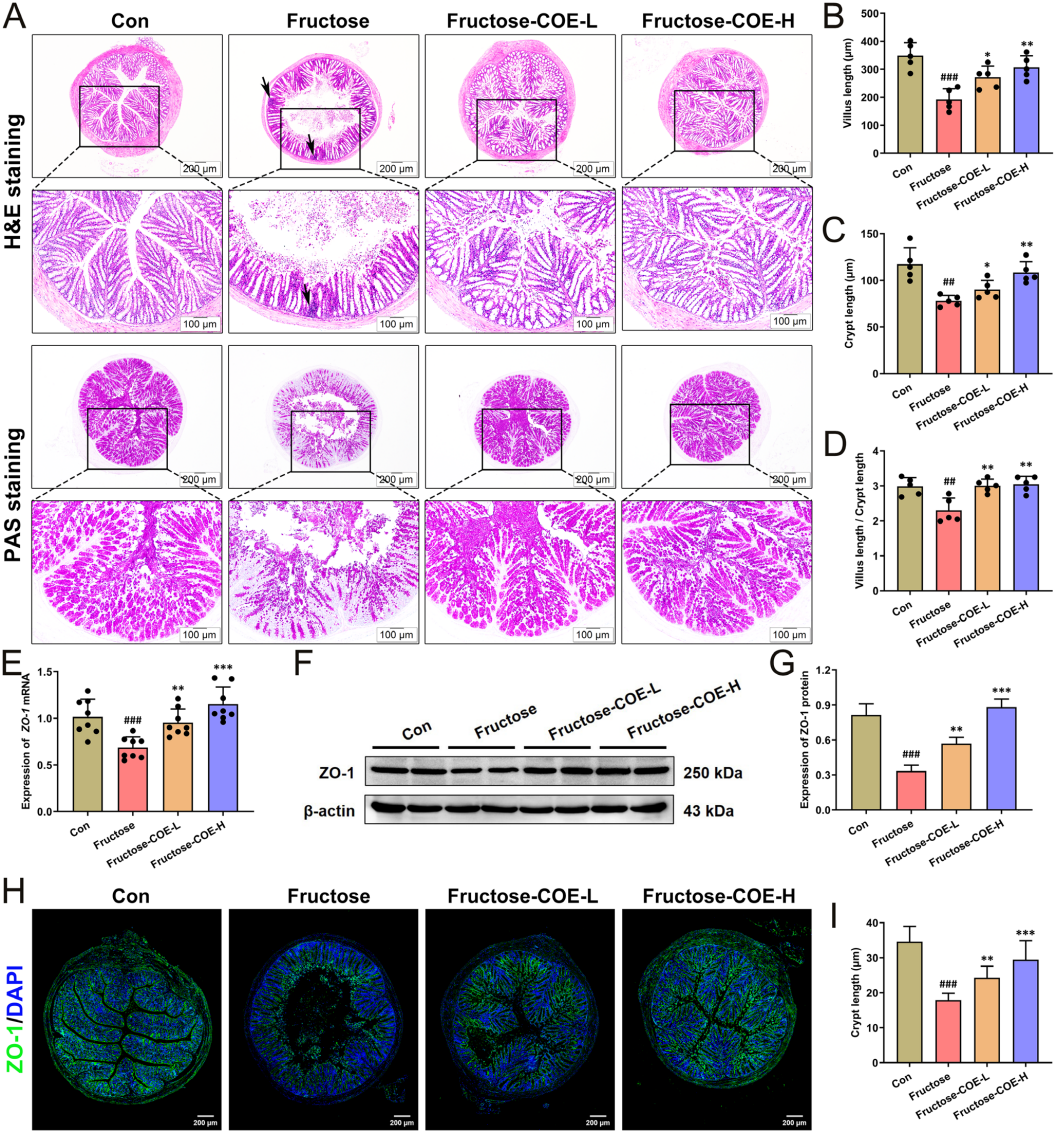
*Cornus officinalis* extract (COE) improves intestinal barrier damage in fructose‐induced mice. (A) Representative images of H&E and PAS staining in the colon tissues (original magnification: 40×, Scale bar: 200 μm; original magnification: 100×, Scale bar: 100 μm). (B–D) Quantitative analysis of villus length (B), crypt depth (C), and the ratio of villus length to crypt depth (D) in the colon tissues (*n* = 5). (E) The mRNA expression levels of *ZO‐1* in the colon tissues (*n* = 8). (F, G) Representative immunoblot images and quantitative analysis of ZO‐1 protein expression (*n* = 3). β‐Actin confirms loading in immunoblotting. (H, I) Representative immunofluorescence images and quantitative analysis of fluorescence intensity for ZO‐1 protein expression (*n* = 3). Mean ± SD, ^##^ *p* < 0.01, ^###^ *p* < 0.001 versus the Con group; *p < 0.05, **p < 0.01, ***p < 0.001 versus the fructose‐induced group. COE‐L and COE‐H represent intragastric administration of *Cornus officinalis* extract at low (100 mg/kg) and high (200 mg/kg) doses, respectively.

Evidence showed that the gut‐liver axis is involved in the etiology of NAFLD (Albillos et al. 2020). In this study, histological staining of the intestine showed that compared with the control group, the colonic villi were heavily broken and arranged disorderly in fructose‐fed mice along with obvious inflammatory infiltration (black arrows) and reduced colonic goblet cells unevenly distributed, while the colonic villi of COE‐treated mice were arranged neatly and tightly and no inflammatory infiltration was seen accompanied by increased colonic goblet cell number (Figure 5A). Meanwhile, the colonic villi length, crypt depth, and the ratio of villus length/crypt depth were decreased in fructose‐fed mice, whereas increased by COE treatment (Figure 5B–D). Moreover, our results showed that the mRNA level and protein expression of important genes related to the tight junction (ZO‐1) were decreased in the colon of fructose‐fed mice, but were increased after COE administration (Figure 5E–G). Immunofluorescence images showed consistent results (Figure 5H,I). Altogether, this demonstrates that COE can protect intestinal barrier integrity in fructose‐fed mice.

### 3.6 COE Attenuated Fructose‐Induced Gut Microbiota Dysbiosis in Mice

**FIGURE 6:**
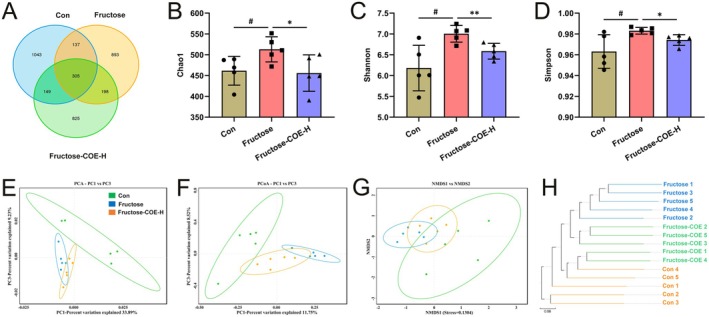
*Cornus officinalis* extract (COE) improves the diversity of gut microbiota in fructose‐fed mice. (A) Venn diagram of operational Taxonomic Units (OUT) number of the gut microbiota in Con or fructose‐fed mice with intragastric administration of either vehicle or COE. (B–D) Statistical histograms of alpha diversity indices for Chao1 (B), Shannon (C), and Simpson (D). (E–G) Principal component analysis (PCA) (E), principal coordinates analysis (PCoA) (F), and non‐metric multidimensional scaling (NMDS) (G) analysis of the gut microbiota. (H) Unweighted pair‐group method with arithmetic mean (UPGMA) analysis of the gut microbiota. Mean ± SD, *n* = 5. ^#^ *p* < 0.05 versus the Con group; *p < 0.05, **p < 0.01 versus the fructose‐induced group. COE‐H represent intragastric administration of *Cornus officinalis* extract at high (200 mg/kg) doses.

Next, we further investigated the effect of COE on alteration in the composition of intestinal microbiota in fructose‐fed mice. 16S rRNA gene sequence analysis demonstrated that there were 1634 operational taxonomic units (OTU) in the control group, 1533 in the fructose‐diet group, and 1477 in the COE‐treated group, among which 305 OTUs were shared (Figure 6A). The Chao1, Shannon, and Simpson diversity indices were used to estimate the alpha diversity of gut flora, and these diversity indices were increased in the gut flora of fructose‐fed mice while decreased by COE treatment (Figure 6B–D). Moreover, principal component analysis (PCA), principal coordinates analysis (PCoA), and non‐metric multidimensional scaling (NMDS), the analysis of β‐diversity, showed that the fructose‐fed group was clearly separated from the other groups, suggesting fructose‐fed led to an obvious gut microbiota composition dysbiosis. However, the COE‐administrated group showed a closer trend toward the control group, demonstrating a positive effect of COE in gut microbiota regulation (Figure 6E–G). In addition, using the Unweighted Pair‐group Method with Arithmetic Mean (UPGMA) to analyze microbial community similarity among the samples, the results also show that the samples in each group can be clearly distinguished (Figure 6H). These results indicate that COE can improve the fructose‐induced altered composition of gut microbiota in mice.

**FIGURE 7:**
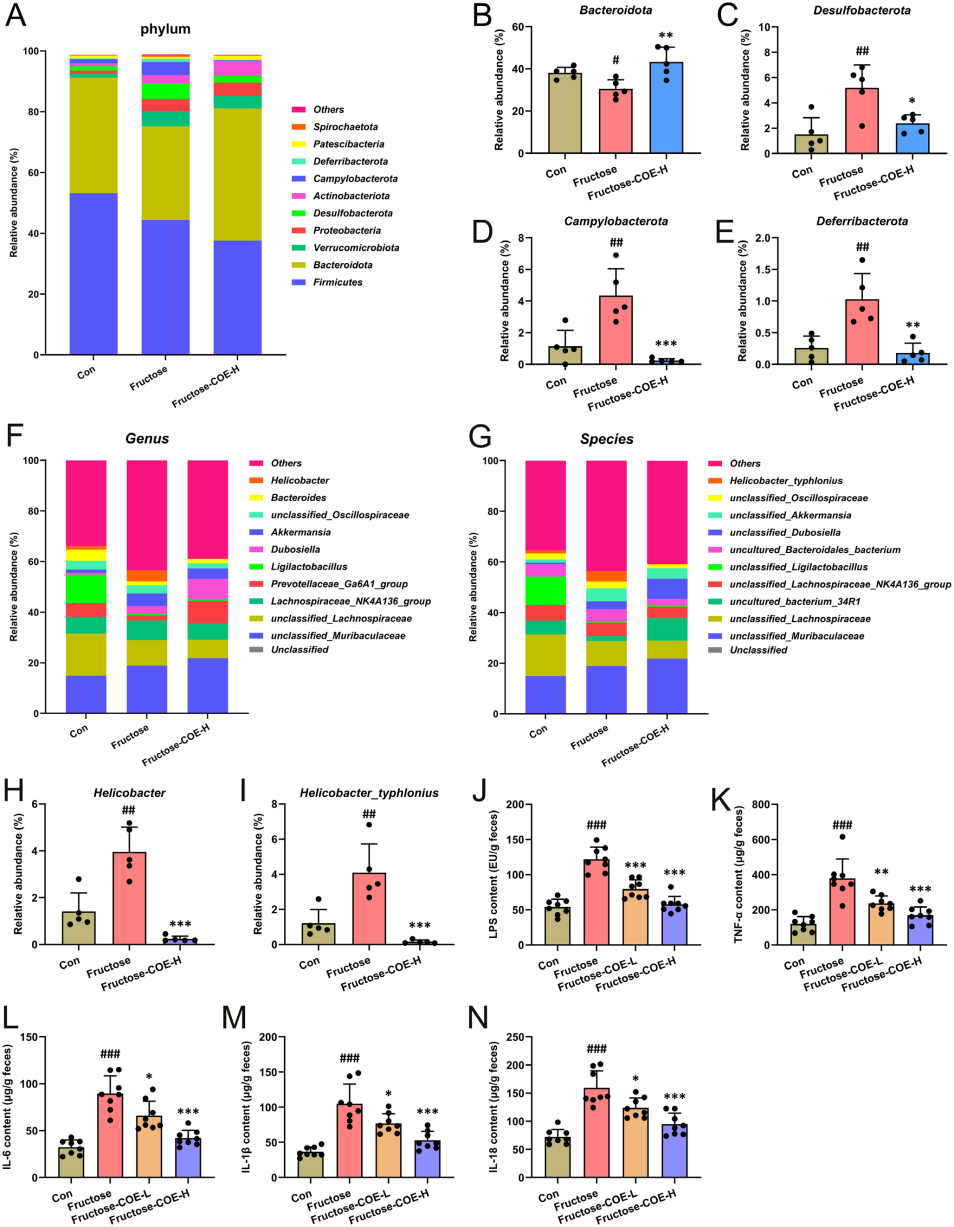
*Cornus officinalis* extract (COE) alleviates gut microbiota disorder in fructose‐fed mice. (A) Histogram of microbial species distribution at the phylum level. (B‐E) Relative abundance of *Bacteroidota* (B), *Desulfobacterota* (C), *Campylobacterota* (D), and *Deferribacterota* (E) at the phylum level (*n* = 5). (F, G) Histogram of microbial species distribution at the genus (F) and species (G) level. (H) Relative abundance of *Helicobacter* at the genus level (*n* = 5). (I) Relative abundance of *Helicobacter_typhlonius* at the genus level (*n* = 5). Contents of the pro‐inflammatory factors LPS, TNF‐α, IL‐6, IL‐1β, and IL‐18 in the feces (*n* = 8). Mean ± SD, ^#^ *p* < 0.05, ^##^ *p* < 0.01, ^###^ *p* < 0.001 versus the Con group; *p < 0.05, **p < 0.01, ***p < 0.001 versus the fructose‐induced group. COE‐H represents intragastric administration of *Cornus officinalis* extract at high (200 mg/kg) doses.

We subsequently analyzed the alterations in the microbiota composition of each group at the phylum level. The results showed that compared with the control group, the relative abundance of *Bacteroidota* was significantly decreased, and the relative abundances of *Desulfobacterota*, *Campylobacterota*, and *Deferribacterota* were increased in the intestine of fructose‐fed mice but could be reversed by COE treatment (Figure 7A–E). At the level of genus and species, it was found that the relative abundances of *Helicobacter* species and *Helicobacter_typhlonius* were increased in the fructose group, while they were decreased in the COE‐treated group (Figure 7F–I). The histogram of the evolutionary branch and LDA value distribution showed that the intestinal flora mainly consisted of *Lactobacillales* and Prevotellaceae in the control group, and *Typhlonius*, *Desulfovibrio*, and Deferribacteraceae under the *Helicobacter* genus in the fructose‐diet group, while the main flora in the COE‐treated group were *Coprostanoligenes* of the *Eubacterium* genus (Figure S2). Furthermore, the contents of pro‐inflammatory cytokines (LPS, TNF‐α, IL‐6, IL‐1β, and IL‐18) in the feces of fructose‐fed mice were also reduced after COE treatment (Figure 7J–N). It indicates that COE is capable of reducing pro‐inflammatory flora‐induced intestinal inflammation and intestinal barrier damage by improving intestinal flora homeostasis.

### 3.7 Transplantation of the Gut Microbiota of Fructose‐Fed Mice Eliminates the Ameliorative Effect of COE in Fructose‐Induced NAFLD

**FIGURE 8:**
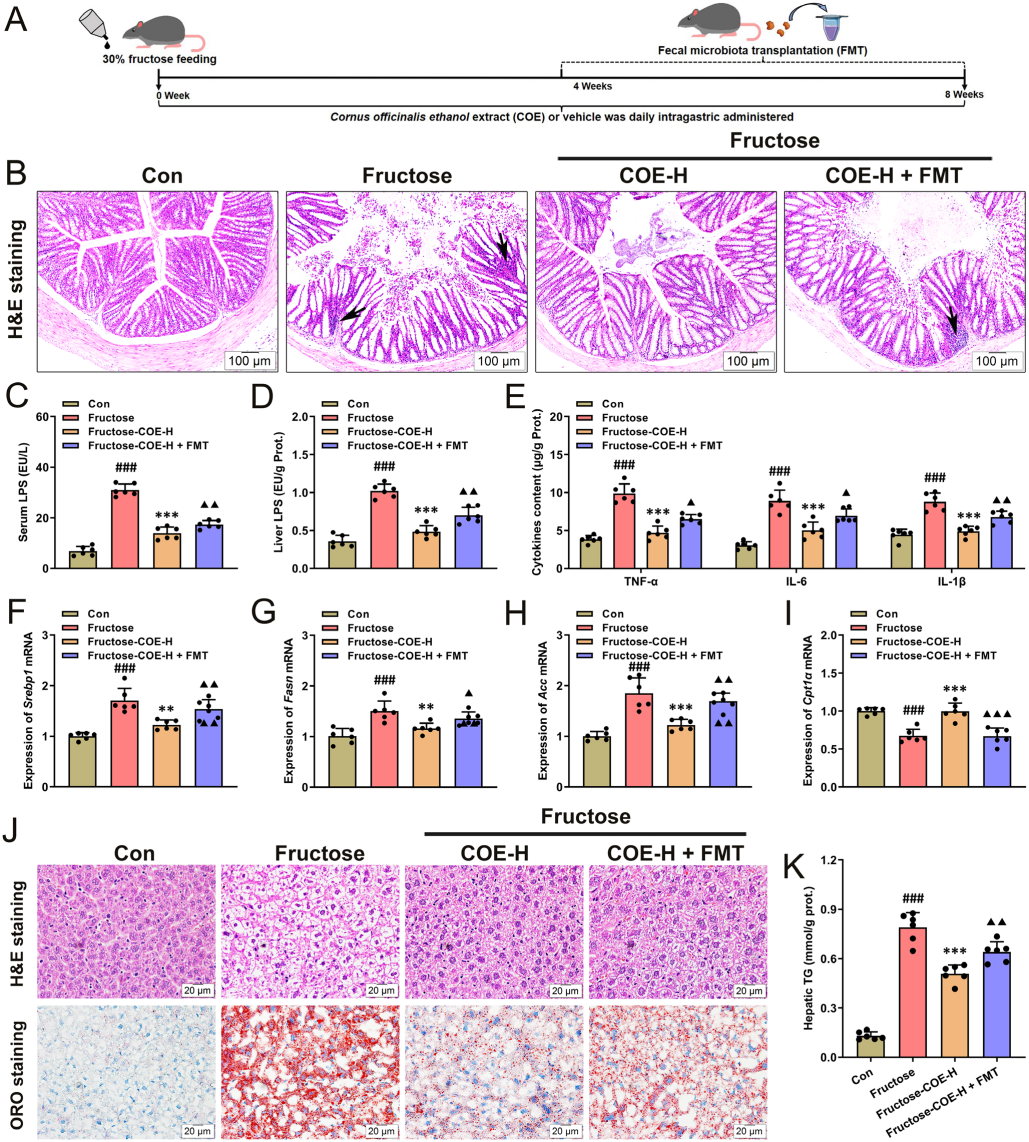
Fecal microbiota transplant impairs the effect of *Cornus officinalis* extract (COE) on improving NAFLD in fructose‐fed mice. (A) Study design. (B) H&E staining of the colon samples in fructose‐fed mice with intragastric administration of either vehicle or COE in the absence or presence of fecal microbiota transplant, respectively (original magnification: 100×, scale bar: 100 μm). (C, D) LPS content in serum (C) and liver (D). (E) Contents of the pro‐inflammatory factors TNF‐α, IL‐6, and IL‐1β in the liver. (F‐I) The mRNA expression levels of *Srebp1* (F), *Fasn* (G), *Acc* (H), and *Cpt1α* (I) in the liver. (J) H&E staining of the liver samples in fructose‐fed mice with intragastric administration of either vehicle or COE in the absence or presence of fecal microbiota transplant, respectively (original magnification: 400×, scale bar: 20 μm). (K) TG content in liver tissue samples. Means ± SD, *n* = 6. ^###^ *p* < 0.001 versus the Con group; **p < 0.01, ***p < 0.001 versus the fructose‐induced group; ^▲^ *p* < 0.05, ^▲▲^ *p* < 0.01, ^▲▲▲^ *p* < 0.001 versus the fructose‐COE‐H group. COE‐H represents intragastric administration of *Cornus officinalis* extract at high (200 mg/kg) doses.

To validate the essential role of intestinal microbiota in the beneficial effect of COE on NAFLD in fructose‐fed mice, we transplanted the fecal microbiota of fructose‐fed mice into COE‐treated mice (Figure 8A). COE was found to be effective in mitigating fructose‐induced intestinal histological damage and inflammatory infiltrates (black arrows), whereas flora transplantation reversed the positive effect of COE (Figure 8B). Meanwhile, serum and liver LPS contents were unregulated in the flora‐transplanted mice along with elevated contents of proinflammatory factors TNF‐α, IL‐6, and IL‐1β (Figure 8C–E). Moreover, the mRNA levels of important lipid metabolic factors (SREBP‐1, FASN, and ACC) were increased along with decreased CPT1α in the flora‐transplanted mice (Figure 8F–I). Of note, the ameliorative effect of COE on fructose‐induced hepatic steatosis was counteracted by flora transplantation, and hepatic TG content was also upregulated (Figure 8J,K). All data reveal that intestinal flora transplantation can partially reverse the inhibitory effect of COE on fructose‐triggered NAFLD in mice.

## 4 Discussion

NAFLD is a central manifestation of hepatic metabolic syndrome that very likely progresses to steatohepatitis, cirrhosis, and even hepatocellular carcinoma with limited treatment due to its complex etiology (Samuel and Shulman 2018). *Cornus officinalis* is an edible pulp of ripe fruit that has been revealed to exert good biological activity in liver protection (Cao et al. 2022; Han et al. 2014; Park et al. 2020, 2021). Previous research has demonstrated the benefits of *C. officinalis* and its processed products in anti‐obesity and reducing hepatic lipid deposition (Cao et al. 2022; Park et al. 2021). However, these available reports are merely exploring the ameliorative effects of *C. officinalis* based on the obesity mice models, and the mechanism accounting for their effects on mitigating cellular lipid accumulation remains poorly elucidated, especially the interaction involving intestinal microbiota homeostasis and hepatocellular steatosis. Herein, this study investigates the ameliorative efficacy of COE in a fructose‐fed mice model and unravels that COE effectively alleviates fructose‐induced NAFLD by restoring intestinal microbiota homeostasis to correct lipid metabolic dysfunction.

Fructose, a carbohydrate mainly obtained from refined sugar and high‐fructose corn syrup in modern diets, especially in the Western world (Stricker et al. 2021), is increasingly regarded as a major driver of the rising morbidity in numerous so‐called lifestyle diseases, such as NAFLD (Ouyang et al. 2008). Traditional academic views suggest that fructose‐induced NAFLD primarily occurs because fructose is rapidly metabolized within hepatocytes by ketohexokinase into fructose 1‐phosphate, eventually giving rise to citrate. Citrate is subsequently converted into acetyl‐CoA by the ATP citrate lyase enzyme (ACLY), then transformed into malonyl CoA, serving as the substrate for lipogenesis (Herman and Samuel 2016; Softic et al. 2016). Furthermore, hepatic fructose metabolism disrupts lipid metabolic homeostasis by enhancing *de novo* lipogenesis and diminishing fatty acid β‐oxidation through altered expression of relevant molecules, ultimately resulting in hepatic lipid accumulation (Softic et al. 2019; Zhao et al. 2020). AMP‐activated protein kinase (AMPK) plays a critical role in integrating metabolic pathways in response to energy demands and exerts protective effects against fructose‐induced hepatic steatosis (Woods et al. 2017). In line with this, our data demonstrated that long‐term high fructose diets induced hepatic steatosis, characterized by TG accumulation and suppressed AMPK activation in mice. Additionally, enhanced lipogenic SREBP‐1/FASN/ACC signaling at both transcriptional and translational levels, along with impaired fatty acid oxidation, further exaggerates hepatic steatosis. Another notable concept proposed in numerous studies is that fructose‐induced liver pathology involves interactions between the gut and liver (Albillos et al. 2020; Febbraio and Karin 2021). A key study published in Nature (2020) suggested that dietary fructose metabolized in the small intestine could be converted into acetate by gut microbiota, triggering lipid synthesis independently of ACLY (Zhao et al. 2020). This finding indicates that the interactions between intestinal functions and the liver in fructose‐driven NAFLD merit further attention. The intestinal mucosal barrier serves as a critical interface for gut–liver interactions by limiting systemic dissemination of microbes and toxins reaching the liver. Unfortunately, fructose overconsumption damages the intestinal epithelial barrier by increasing intestinal permeability, driven by intestinal inflammation and endoplasmic reticulum stress (Cho et al. 2021; Kawabata et al. 2019). Consequently, this facilitates systemic portal influx of gut‐derived LPS and microbial metabolites into the liver (Cho et al. 2021; Todoric et al. 2020). LPS subsequently initiates inflammatory cascades through macrophage TLR4 signaling, promoting NAFLD progression (Krishnan et al. 2018; Lambertz et al. 2017). Our findings showed that the chronic fructose diet destructed the intestinal epithelial mucosa and reduced the tight junction protein ZO‐1, a key player in intestinal mucosal barrier integrity. Moreover, gut‐derived LPS contents were increased in fructose‐fed mice and flowed to the liver via the portal vein, leading to the activation of the LPS‐stimulated inflammatory response, while these were improved by COE treatment. Besides motivating inflammation, the generation of proinflammatory cytokines such as TNF‐α also functioned as a potent lipid metabolism regulator, accelerating hepatocyte lipid deposition and steatosis (Chen et al. 2009; De Taeye et al. 2007; Endo et al. 2007). Consistently, excessive lipid accumulation could further promote TLR4/NF‐κB signaling to trigger the leakage of proinflammatory cytokines (TNF‐α, IL‐6, IL‐1β, and IL‐18), thus exacerbating the vicious cycle of inflammatory responses in the liver to aggravate fatty liver progression (Fei et al. 2020). Consistently, this study found that COE inhibited hepatic inflammatory signaling activation in fructose‐fed mice, which may be related to the fact that COE improved intestinal function to inhibit LPS‐induced inflammatory response.

Notably, microbial dysbiosis is considered primarily responsible for intestinal deterioration, and maintaining microbial homeostasis is critical for preserving liver health (Aron‐Wisnewsky et al. 2020). Therefore, determining gut microbiota composition is essential to investigate the causal relationships and potential pathogenic links between dysbiosis and NAFLD. Given the improving effect of COE on intestinal permeability, inflammation, and liver metabolism, we further analyzed the composition of intestinal microbiota in different groups to identify the possible involvement of COE in regulating intestinal bacterial composition. Our results showed a higher relative abundance of pro‐inflammatory bacteria (*Helicobacter_typhlonius*) in the fructose‐fed mice along with the generation of a large number of proinflammatory cytokines and intestinal barrier damage, whereas COE treatment reduced the relative abundance of *Helicobacter_typhlonius* and attenuated intestinal inflammation. *Helicobacter_typhlonius* is known as a species of *Helicobacter pylori* that triggers intestinal dysbiosis and gut inflammation and plays a vital role in the progression of intestinal inflammatory diseases as an inducer of enterocolitis disease models (Bostick et al. 2019; Franklin et al. 2001). Research has revealed that *Helicobacter_typhlonius* infection could evoke excessive production of TNF‐α, a key disease trigger that promotes the progression of colitis (Chai et al. 2017; Hale et al. 2007; Powell et al. 2012). It is suggested that COE may protect the intestinal barrier and function by reducing intestinal *Helicobacter_typhlonius* colonization to attenuate the intestinal inflammatory response in fructose‐diet mice. Moreover, transplanting the gut microbiota isolated from fructose‐fed mice into COE‐treated mice depletion led to a partial reversal of its improvements in intestinal injury, liver inflammation, and lipid metabolism disorder, further indicating the central role of gut microbiota in delaying the progression of NAFLD by COE treatment. Phytochemical studies have reported that *C. officinalis* contains various types of chemical constituents including loganin, morroniside, cornuside, sweroside, gallic acid, and ursolic acid, etc. (He et al. 2016; Huang et al. 2018). In particular, loganin and morroniside, which are indicator components of *C. officinalis* , have been shown to improve metabolic disorders in obese mice (Jang et al. 2023; Park et al. 2009; Xu et al. 2022; Yamabe et al. 2010). Hence, COE may improve NAFLD through constraining lipid metabolism homeostasis and mitigating intestinal bacterial dysbiosis by its multiple active components.

In summary, this study reveals that COE is effective in improving fructose‐induced NAFLD by constraining lipid metabolism homeostasis and mitigating intestinal bacterial dysbiosis‐modulated inflammatory response, especially emphasizing the essential role of microbiota homeostasis in this therapeutic course. The current study provides a preclinical theoretical basis for supplementation of the medicinal food *Cornus officinalis* as an effective strategy for the treatment of NAFLD.

## Author Contributions

**Liang Chen:** conceptualization (equal), data curation (equal), investigation (equal), writing – original draft (equal). **Yingying Song:** conceptualization (equal), data curation (equal), investigation (equal), writing – original draft (equal). **Yurou Huang:** conceptualization (equal), data curation (equal), investigation (equal), writing – original draft (equal). **Junjie Hu:** methodology (equal). **Yan Meng:** formal analysis (equal), software (equal), visualization (equal). **Ming Yuan:** formal analysis (equal), software (equal), visualization (equal). **Guohua Zheng:** project administration (equal). **Xuanbin Wang:** conceptualization (equal), writing – review and editing (equal). **Cong Zhang:** conceptualization (equal), funding acquisition (equal), writing – original draft (equal). **Zhenpeng Qiu:** conceptualization (equal), funding acquisition (equal), writing – review and editing (equal).

## Conflicts of Interest

The authors declare no conflicts of interest.

## Supporting information


Data S1.

## Data Availability

The data are available from the corresponding author on reasonable request.
