# Supplementary material for: Cornus officinalis Extract Ameliorates Fructose‐Induced Hepatic Steatosis in Mice by Sustaining the Homeostasis of Intestinal Microecology and Lipid Metabolism
Source: Food Sci Nutr. 2025 Jun 11;13(6):e70425. doi: 10.1002/fsn3.70425 (PMC12152365; doi:10.1002/fsn3.70425)
Supplement: Supplementary file 1 — Data S1. [file FSN3-13-e70425-s001.pdf]

Supplementary Materials

Cornus officinalis **extract ameliorates fructose-induced hepatic steatosis in mice by sustaining the homeostasis of intestinal microecology and lipid metabolism**

Supplementary Figures

Supplementary Figure S1. Qualitative analysis of 5 representative compounds (morroniside, loganin, cornuside I, gallic acid, and magnolin) in *Cornus officinalis* extract (COE) was characterized by HPLC analysis, respectively. (A) HPLC chromatograms of COE; (B) HPLC chromatograms of morroniside, loganin, cornuside I, gallic acid, and magnolin standards. 1: morroniside, 2: loganin, 3: cornuside I, 4: gallic acid, 5: magnolin.

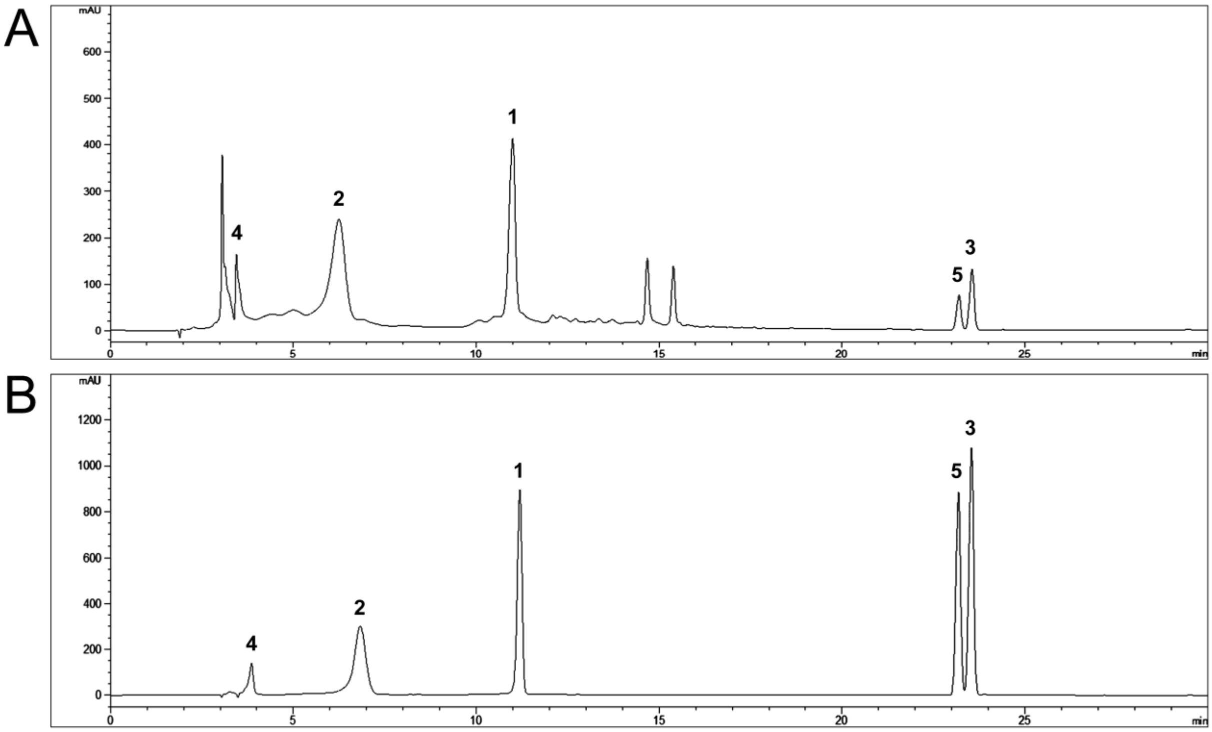

Supplementary Figure S2. Analysis of intestinal microbial community diversity by

LEfSe. The histogram of the evolutionary branch (A) and LDA value distribution (B) showed that the intestinal flora mainly consisted of *Lactobacillales* and *Prevotellaceae* in the control group, and *Typhlonius*, *Desulfovibrio*, and *Deferribacteraceae* under the *Helicobacter* genus in the fructose-diet group, while the main flora in the *Cornus officinalis* extract (COE)-treated group were *Coprostanoligenes* of the *Eubacterium* genus.

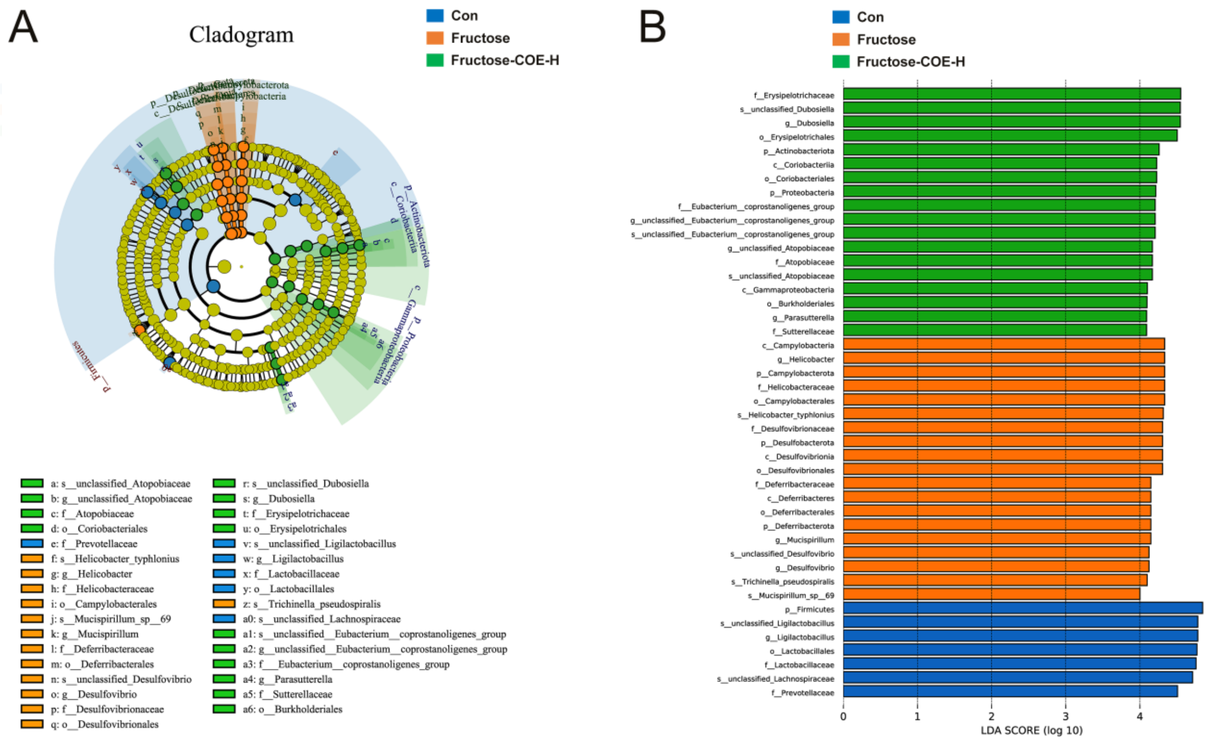

Supplementary Tables

Supplementary Table S1. List of the primary antibodies used for Western blot (WB) analysis, immunohistochemistry (IHC), and immunofluorescence (IF) analysis.

Protein	Antibody (and catalog number)	Application
ACC	Rabbit monoclonal (3676)	WB; IHC †
AMPKα	Rabbit monoclonal (5831)	WB †
CPT1α	Rabbit polyclonal (15184-AP)	WB; IHC #
FASN	Rabbit monoclonal (3180)	WB; IHC †
p-AMPKα (Thr172)	Rabbit monoclonal (2535)	WB; IF †
SREBP-1	Rabbit polyclonal (TA6283)	WB; IHC *
ZO-1	Rabbit polyclonal (A0659)	WB; IF §
β-actin	Mouse monoclonal (66009-1-Ig)	WB #
HRP Rabbit Anti-Goat IgG (H+L)	AS029	WB; IHC §
Cy3 Rabbit Anti-Goat IgG (H+L)	AS015	IF §

† Provided by Cell Signaling Technology Inc. (Danvers, MA)

Provided by Proteintech (Wuhan, China)

§ Provided by ABclonal (Wuhan, China)

* Provided by Abmart (Shanghai, China)

Supplementary Table S2. Sequences of primers used in real-time quantitative PCR (RT-qPCR)

Genes	Sequences (5' to 3')	
GAPDH	Forward: TCAATGAAGGGGTCGTTGAT	Reverse: CGTCCCGTAGACAAAATGGT
Srebp1	Forward: TGACCCGGCTATTCCGTGA	Reverse: CTGGGCTGAGCAATACAGTTC
Fasn	Forward: CCAAGCAGGCACACACAA	Reverse: CACTCACACCCACCCAGA
Acc	Forward: GATGAACCATCTCCGTTGGC	Reverse: GACCCAATTATGAATCGGGAGTG
Cpt1α	Forward: GGCCATCTGTGGGAGTATGT	Reverse: ACTGTAGCCTGGTGGGTTTG
TNF-α	Forward: CAGGCGGTGCCTATGTCTC	Reverse: CGATCACCCCGAAGTTCAGTAG
IL-1β	Forward: GAAATGCCACCTTTTGACAGTG	Reverse: TGGATGCTCTCATCAGGACAG
IL-6	Forward: TAGTCCTTCCTACCCCAATTTCC	Reverse: TTGGTCCTTAGCCACTCCTTC
IL-18	Forward: AGTGAACCCCAGACCAGACT	Reverse: TCAGGTGGATCCATTTCCTCAA
ZO-1	Forward: ACCCGAAACTGATGCTGTGGATAG	Reverse: AAATGGCCGGGCAGAACTTGTGTA

Supplementary Table S3. List of the major pharmacodynamic components in *Cornus officinalis* extract (COE) identified by UPLC-Q-TOF-MS analysis.

Table S3. List of the major phytochemical constituents in COE

Compounds	Molecular formula	Theoretical m/z	Detection m/z
Ammodendrine	$C_{12}H_{20}N_2O$	208.1580	208.1576
Autumnolide	$C_{15}H_{20}O_5$	280.1310	280.1308
Caulophylline	$C_{12}H_{16}N_2O$	204.1263	204.1255
Ceanothine E	$C_{34}H_{40}N_4O_4$	568.3050	568.3057
Coriose	$C_7H_{14}O_7$	210.0740	210.0740
Cornuside I	$C_{24}H_{30}O_{14}$	542.4870	542.4850
Cucurbic acid	$C_{12}H_{20}O_3$	212.1410	212.1413
Cyperolone	$C_{15}H_{24}O_2$	236.1780	236.1775
Echioidin	$C_{22}H_{22}O_{10}$	446.1213	446.1213
Echujin	$C_{42}H_{66}O_{17}$	842.4300	842.4295
Embelin	$C_{17}H_{26}O_4$	294.1831	294.183
Eudesmin	$C_{22}H_{26}O_6$	386.1729	386.1724
Frangulin B	$C_{20}H_{18}O_9$	402.0950	402.0952
Gallic acid	$C_7H_6O_5$	170.1200	170.1300
Gardenoside	$C_{17}H_{24}O_{11}$	404.1319	404.1323
Guttiferone A	$C_{38}H_{50}O_6$	602.3610	602.3631
Homodolicholide	$C_{29}H_{48}O_6$	492.3450	492.3429
Hordatine A	$C_{28}H_{38}N_8O_4$	550.3020	550.3033
Isoprunetin-7-O-β-D-glucopyranoside	$C_{22}H_{22}O_{10}$	446.1213	446.1201
Kaempferol-3-(2''-p-coumarylglucoside)	$C_{30}H_{26}O_{13}$	594.1370	594.1375
Linoleic acid	$C_{18}H_{32}O_2$	280.2402	280.2399
Loganin	$C_{17}H_{26}O_{10}$	390.1526	390.1526
Magnolin	$C_{23}H_{28}O_7$	416.4600	416.4380
Obtusifolin-2-glucoside	$C_{22}H_{22}O_{10}$	446.1210	446.1224
Panaxatriol	$C_{30}H_{52}O_4$	476.3866	476.3860
Quebrachitol	$C_7H_{14}O_6$	194.0790	194.0786
Sedoheptulose	$C_7H_{14}O_7$	210.0740	210.0743
Theaflavin digallate	$C_{43}H_{32}O_{20}$	868.1487	868.1488
Tiliroside	$C_{30}H_{26}O_{13}$	594.1373	594.1366
Vernoflexuoside	$C_{21}H_{28}O_8$	408.1780	408.1784
α-Linolenic acid	$C_{18}H_{30}O_2$	278.2246	278.2249
